# Supplementary material for: University students and staff able to maintain low daily contact numbers during various COVID-19 guideline periods
Source: Epidemiol Infect. 2021 Aug 10;149:e169. doi: 10.1017/S0950268821001618 (PMC8365047; doi:10.1017/S0950268821001618)
Supplement: Supplementary file 1 [file hygsup.zip › S0950268821001618sup002.pdf]

HIDDEN FIELD
Survey start date and time - taken from participant's device. ______________________________

CON-QUEST (COroNavirus QUESTionnaire): Contact questionnaire on patterns and behaviour in University of Bristol staff and students during the COVID-19 pandemic

Help control COVID-19 Thank you for taking this survey. In doing so, you will be contributing to research into how COVID-19 spreads. Social contact patterns are important for understanding the spread of COVID-19. We are conducting this survey at the University of Bristol to measure social contact patterns in a university setting, as well as other factors important in the transmission of COVID-19. Instructions You must be over 18 years of age to complete this survey and a member of staff or a student at the University of Bristol. The survey is anonymous, and you can opt out at any time by closing the survey. We are unable to remove submitted answers due to the anonymous nature of the survey. Therefore, submission of pages within the survey will be taken as consent that you are happy for the data you have provided to be used for research purposes. Your anonymised data from this questionnaire will be made available publicly through a data repository and through publications to aid with the COVID-19 public health response. As lockdown eases, we will capture how contact patterns and behaviour change over time. If you are happy to be contacted again, you will be able to provide an email address at the end of the survey. Your email address will only be stored for the sole purpose of contacting you about completing this survey in the future. Your email address will not be linked to the information you submit in the questionnaire - your data will remain anonymous. Your information will be kept in accordance with the Data Protection Act. Thank you for completing CON-QUEST! If you have any questions about the survey, then please contact us on: uob-conquest-project@bristol.ac.uk
You can read about the survey and find latest results on the CONQUEST website: bris.ac.uk/con-quest This survey is funded by the Elizabeth Blackwell Institute for Health Research at the University of Bristol.

Background information

HIDDEN FROM SURVEY
Date and time started (taken from user's device)

____

The first part of this survey will collect background information about you so that we can understand how representative our survey respondents are compared to the rest of the university population. You will not need to complete this section again if you fill out future versions of the survey.

Age

____
(Years)

What is your gender?

◯ Female
◯ Male
◯ Other
◯ Prefer not to say

Please specify your gender if you wish __________________________________

How would you describe your ethnicity?

◯ White
◯ Mixed/multiple ethnic groups
◯ Asian/Asian British
◯ Black /African/ Caribbean/Black British
◯ Other ethnic group
◯ Prefer not to say

◯ English / Welsh / Scottish / Northern Irish / British
◯ Irish
◯ Gypsy or Irish traveller
◯ any other white

Please tell us your ethnic group

____

◯ White and Black Caribbean
◯ White and Black African
◯ White and Asian
◯ Any other mixed/multiple ethnic background

Please tell us your ethnic group

____

◯ Indian
◯ Pakistani
◯ Bangladeshi
◯ Chinese
◯ Any other Asian background

Please tell us your ethnic group

____

◯ African
◯ Caribbean
◯ Any other Black/African/Caribbean background

Please tell us your ethnic group

____

◯ Arab
◯ Any other ethnic group

Please tell us your ethnic group

____

Are you pregnant?

◯ Yes
◯ No
◯ Not sure

Are you at a higher risk of severe illness if you catch coronavirus?
(see definitions on the NHS website:
https://www.nhs.uk/conditions/coronavirus-covid-19/people-at-higher-risk/whos-at-higher-risk-from-coronavirus/)

◯ Yes, I was sent a letter from the government saying that I am clinically extremely vulnerable (high risk)
◯ Yes, I am in a clinically vulnerable group (moderate risk)
◯ No
◯ Don't know
◯ Other

Other - please explain:

____

Are you currently living with someone who is shielding?

- ◯ Yes
- ◯ No

Are you:
(If you are both then please tick both options)

- ☐ a student?
- ☐ a member of staff?

- ◯ Undergraduate
- ◯ Postgraduate

What is your main faculty/department

- ◯ Arts
- ◯ Engineering
- ◯ Health Sciences
- ◯ Life Sciences
- ◯ Science
- ◯ Social Sciences and Law
- ◯ Other

Other - please specify

____

School

- ◯ School of Arts
- ◯ School of Humanities
- ◯ School of Modern Languages
- ◯ Centre for Academic Language and Development
- ◯ Centre for Innovation

School

- ◯ School of Computer Science, Electrical and Electronic Engineering, and Engineering Mathematics
- ◯ School of Civil, Aerospace and Mechanical Engineering

School

- ◯ Bristol Dental School
- ◯ Bristol Medical School
- ◯ Bristol Veterinary School
- ◯ Centre for Health Sciences Education

School

- ◯ School of Biological Sciences
- ◯ School of Biochemistry
- ◯ School of Cellular and Molecular Medicine
- ◯ School of Physiology, Pharmacology and Neuroscience
- ◯ School of Psychological Science

School

◯ School of Chemistry
◯ School of Earth Sciences
◯ School of Geographical Sciences
◯ School of Mathematics
◯ School of Physics

School

◯ School of Education
◯ School for Policy Studies
◯ School of Economics, Finance and Management
◯ School of Sociology, Politics and International Studies
◯ University of Bristol Law School

Which academic year of your degree are you currently in (e.g. Year 1 of 4)?

Year

____

of

____

Please check - year of study is greater than total number of years!

Year of study

Part time?

◯ Yes
◯ No
◯ Rather not say

What is your job type at the University?

◯ Clinical Academic (e.g. Clinical Lecturer)
◯ Operational Services (e.g. Cleaner, Porter)
◯ Professional/Administrative Services (e.g. Administrator, Director, Manager, Adviser)
◯ Research and Teaching (e.g. Lecturer, Research Associate)
◯ Technical Services (e.g. Research Technician)

Which faculty, directorate or institution are you part of?
(if more than one then please select your main one)

◯ Alumni Campaigns and Relations
◯ Campus Division
◯ Communications and PR
◯ Estates
◯ Faculty of Arts
◯ Faculty of Engineering
◯ Faculty of Health Sciences
◯ Faculty of Life Sciences
◯ Faculty of Science
◯ Faculty of Social Sciences and Law
◯ Finance
◯ HR
◯ Information Technology
◯ Legal services
◯ Library services
◯ Planning and Projects
◯ Research and Enterprise Development
◯ Residential and Hospitality
◯ Secretarial and PA
◯ Senior Management Team
◯ Sports Exercise and Health
◯ Student administration, recruitment and services
◯ Other

Which school are you a member of?

◯ School of Arts
◯ School of Humanities
◯ School of Modern Languages
◯ Centre for Academic Language and Development
◯ Centre for Innovation

Which school are you a member of?

◯ School of Computer Science, Electrical and Electronic Engineering, and Engineering Mathematics
◯ School of Civil, Aerospace and Mechanical Engineering

Which school are you a member of?

◯ Bristol Dental School
◯ Bristol Medical School
◯ Bristol Veterinary School
◯ Centre for Health Sciences Education

Which school are you a member of?

◯ School of Biological Sciences
◯ School of Biochemistry
◯ School of Cellular and Molecular Medicine
◯ School of Physiology, Pharmacology and Neuroscience
◯ School of Psychological Science

Which school are you a member of?

◯ School of Chemistry
◯ School of Earth Sciences
◯ School of Geographical Sciences
◯ School of Mathematics
◯ School of Physics

Which school are you a member of?

◯ School of Education
◯ School for Policy Studies
◯ School of Economics, Finance and Management
◯ School of Sociology, Politics and International Studies
◯ University of Bristol Law School

Does the nature of your role mean that the majority of your work must be done on location at the University (i.e. working from home full time is not a feasible option for you)?

◯ Yes - the majority of my work takes place in a laboratory
◯ Yes - my role involves maintaining or monitoring University buildings or grounds
◯ Yes - other reason
◯ No
◯ Not sure

Please specify reason

____

Contacts where you live and work

This section will help us understand more about the contacts that you have in the place that you live.

Where do you currently live during term-time?

◯ Hall of residence (Catered)
◯ Hall of residence (Self- Catered)
◯ Shared house/flat
◯ With immediate family (parents/siblings/children)
◯ Live alone
◯ Other

Other - please specify

____

Has your term time residence changed as a result of COVID-19?

◯ Yes ◯ No

Where would you usually live during term-time?

◯ Hall of residence (Catered)
◯ Hall of residence (Self- Catered)
◯ Shared house/flat
◯ With immediate family (parents/siblings/children)
◯ Live alone
◯ Other

Other - please specify

____

Where do you usually live out of term time?

◯ The same place as during term time
◯ Hall of residence (Catered)
◯ Hall of residence (Self- Catered)
◯ Shared house/flat
◯ With immediate family (parents/siblings/children)
◯ Live alone
◯ Other

Other - please specify

____

This question is hidden from the survey
Where do you currently live (now that social distancing has been imposed and the University buildings have closed)?

◯ The same place as during term time
◯ Different halls of residence (Catered)
◯ Different halls of residence (Self- Catered)
◯ Different shared house/flat
◯ With immediate family (parents/siblings/children)
◯ Live alone
◯ Other

Other - please specify

____

How many people in the following age categories are currently living with you (including yourself)?
If you are living at more than one address currently, then please answer this question for the address where you spend most of your time.

If you live in a hall of residence, we are asking about your living circle (https://www.bristol.ac.uk/students/your-studies/study-2020/living-in-residences/)

Age 0-4:

____

Age 5-17:

____

Age 18-24:

____

Age 25-44:

____

Age 45-64:

____

Age 65-80:

____

Age 81+

____

*This question is hidden from the survey*Is your household made up of different people as a result of the COVID-19 pandemic

◯ Yes ◯ No ◯ Prefer not to say

This question is hidden from the survey
How many people lived in your previous household (including yourself) in the following age categories?

This question is hidden from the survey
If you lived in a Halls of residence, we are asking about those who you shared a bathroom/kitchen/living space/dining room with - choose the area which you share with the maximum number of people.

*This question is hidden from the survey*Age 0-4:

____

*This question is hidden from the survey*Age 5-17:

____

*This question is hidden from the survey*Age 18-24:

____

*This question is hidden from the survey*Age 25-44:

____

*This question is hidden from the survey*Age 45-64:

____

*This question is hidden from the survey*Age 65-80:

____

*This question is hidden from the survey*Age 81+

____

Are you currently in a support bubble with another household? (If you are not sure what a support bubble is, then you can find an official description here:
https://www.ageuk.org.uk/information-advice/coronavirus/coronavirus-guidance/support-bubbles/)

◯ Yes ◯ No

How many people in the following age categories are in your support bubble (not including you or those in your household)?

Age 0-4:

____

Age 5-17:

____

Age 18-24:

____

Age 25-44:

____

Age 45-64:

____

Age 65-80:

____

Age 81+

____

*This question is hidden from the survey*Due to COVID-19 have you, or would you, consider taking the following action when the University is open?

This question is hidden from the survey
Avoid lectures attended by many people:

◯ I have done this
◯ I have not done this, but I intend to do so
◯ I have not done this and do not intend to do so

This question is hidden from the survey
Work from home where possible :

◯ I have done this
◯ I have not done this, but I intend to do so
◯ I have not done this and do not intend to do so

This question is hidden from the survey
Avoid using shared facilities:

◯ I have done this
◯ I have not done this, but I intend to do so
◯ I have not done this and do not intend to do so

This question is hidden from the survey
Wear a face mask or covering when at the University:

◯ I have done this
◯ I have not done this, but I intend to do so
◯ I have not done this and do not intend to do so

This question is hidden from the survey
Avoid as much contact as possible by not attending any face-to-face activities on campus:

◯ I have done this
◯ I have not done this, but I intend to do so
◯ I have not done this and do not intend to do so

This question is hidden from the survey
Reduce or eliminate social activities involving more than two people:

◯ I have done this
◯ I have not done this, but I intend to do so
◯ I have not done this and do not intend to do so

*This question is hidden from the survey*Are you currently using any COVID-19 symptom tracker or contact tracing apps ?

◯ Yes, and I would consider using similar apps in the future.
◯ Yes, but I would not consider using similar apps in the future.
◯ No, but I would consider using similar apps in the future.
◯ No, and I would not consider using similar apps in the future.

Would you be willing to be tested for current or previous COVID-19 infection using (tick all that apply):

☐ Saliva
☐ Throat swab
☐ Blood test
☐ Finger prick
☐ None of these
